# HPA-UNet-LSNet: An LSNet-based U-Net with hybrid pooling attention for accurate segmentation of *Haloxylon ammodendron* crowns from UAV RGB imagery

**DOI:** 10.1371/journal.pone.0350455

**Published:** 2026-06-12

**Authors:** Dongze Li, Xuefeng Yang, Yingnan Li, Ting Liang

**Affiliations:** 1 College of Geographical Sciences and Tourism, Xinjiang Normal University, Urumqi, Xinjiang, China; 2 Xinjiang Laboratory of Lake Environment and Resources in Arid Regions, Urumqi, Xinjiang, China; 3 Office of Academic Research, Xinjiang Normal University, Urumqi, China; Federal University Otuoke, NIGERIA

## Abstract

Accurate segmentation of *Haloxylon ammodendron* crowns from UAV RGB imagery remains challenging in desert environments because of sparse crown distribution, weak crown–background contrast, and interference from sandy soil and co-occurring shrubs. To address this problem, this study developed **HPA-UNet-LSNet**, an enhanced U-Net framework that replaces the original encoder with LSNet and introduces hybrid pooling attention (HPA) for feature fusion. On the independent test set, HPA-UNet-LSNet achieved a Precision of 0.8890, a Recall of 0.9198, an F1-score of 0.9041, and an mIoU of 0.8456. Compared with the baseline U-Net, it reduced false positives from 454 ± 53 to 267 ± 18 and false negatives from 224 ± 11 to 185 ± 10. The improvement was especially evident for small crowns, where the F1-score increased from 0.7318 ± 0.0179 to 0.7611 ± 0.0102, and the mIoU increased from 0.6498 ± 0.0045 to 0.6929 ± 0.0089. Grad-CAM results further showed more concentrated responses over crown regions and relatively reduced activation in irrelevant background areas. Overall, HPA-UNet-LSNet provides an effective and practical RGB-based solution for *Haloxylon ammodendron* crown segmentation in desert environments.

## Introduction

Against the backdrop of intensifying global climate change and increasing desertification, dynamic monitoring and ecological protection of vegetation in arid regions have become increasingly important for maintaining regional carbon balance and ecological security [1,2]. *Haloxylon ammodendron*, an extremely drought-tolerant shrub of the genus *Haloxylon* in the Amaranthaceae family, is a dominant species in desert ecosystems of northwestern China. Due to its strong tolerance to drought and saline-alkaline conditions, it plays a crucial role in windbreak and sand fixation, soil conservation, and maintaining the regional carbon-oxygen balance [3,4]. However, under the combined influence of overgrazing, deforestation, and climate change, natural *Haloxylon ammodendron* stands in areas such as the Junggar Basin of the Xinjiang Uygur Autonomous Region have experienced degradation and local dieback, reducing the ecological service value of desert ecosystems [5,6]. Traditional field-based monitoring mainly relies on visual interpretation and manual investigation, which are labor-intensive, subjective, and difficult to scale for large-area, high-precision dynamic vegetation monitoring [7,8].

The development of UAV remote sensing has provided a new opportunity for desert vegetation monitoring. High-resolution RGB imagery acquired by UAV platforms enables refined observation of *Haloxylon ammodendron* canopies [9,10]. In recent years, deep learning has demonstrated substantial advantages in remote sensing image analysis. Object detection models, such as the YOLO series, can efficiently localize multiple targets [11,12]; semantic segmentation models, such as U-Net, can provide pixel-level category prediction [13,14]; and instance segmentation models, such as Mask R-CNN, can generate pixel-wise masks for individual tree crowns [15,16].

Neural-network-based approaches have shown promising performance in identifying individual trees in sparse forest environments [17]. For instance, Weinstein et al. [18] detected individual tree crowns in complex RGB imagery with an average accuracy of 85.3%. Paul et al. [19] achieved an F1-score of 0.914 for shade-tree detection using deep convolutional networks. Hao et al. [20] reported an F1-score of 0.85 for detecting young Chinese fir using Mask R-CNN, and Ji et al. [21] achieved F1-scores ranging from 85.7% to 87.4% for multiple tree species using BlendMask. However, accurately segmenting *Haloxylon ammodendron* crowns remains difficult due to their sparse distribution, relatively weak structural continuity, spectral similarity to sandy backgrounds, and interference from co-occurring shrubs.

Recent advances in backbone design have provided new possibilities for improving feature extraction in challenging segmentation tasks. The LSNet series [22], inspired by the “See Large, Focus Small” strategy, combines large-kernel perception with small-kernel aggregation to efficiently capture both broad contextual cues and fine-grained local details. This design is well suited to UAV RGB crown segmentation, where effective discrimination requires both global contextual awareness and sensitivity to weak local crown structures.

Capturing broader contextual information is also essential for distinguishing sparse crowns from heterogeneous desert backgrounds. However, directly applying standard global self-attention to high-resolution imagery is computationally expensive, whereas conventional convolution mainly emphasizes local structures. To better integrate local and contextual information, Chen et al. [23] proposed a synergistic CNN-Transformer network with pooling attention fusion. Its hybrid pooling attention (HPA) module enhances feature interaction by combining spatial attention with multi-scale aggregation, thereby improving the joint use of local details and broader contextual cues.

The Junggar Basin in the Xinjiang Uygur Autonomous Region is a representative desert ecosystem and a core region of the Gurbantunggut Desert. Its ecological stability is crucial for regional ecological security [24,25]. Existing studies on *Haloxylon ammodendron* have primarily focused on ecological processes, including soil improvement, water-use strategies, and carbon sequestration dynamics [4,26–28]. In contrast, research on precise canopy monitoring and information extraction using advanced remote sensing techniques remains limited.

Related challenges, such as class imbalance, small-target detection, and complex background interference, have also been discussed in other machine-learning and image-analysis studies [29–34]. These observations further suggest that robust feature representation and effective foreground-background discrimination are broadly important in challenging visual recognition tasks.

Nevertheless, crown segmentation of *Haloxylon ammodendron* in UAV-based desert imagery remains particularly difficult. Existing methods still suffer from insufficient segmentation accuracy, difficulty in delineating crown boundaries due to sparse branching structures and spectral confusion with sandy backgrounds, confusion with co-occurring shrubs such as *Tamarix*, and frequent missed detections of small individuals such as seedlings. Consequently, current approaches remain inadequate for fine-scale and dynamic monitoring in arid environments.

To address these challenges, we propose HPA-UNet-LSNet, a task-oriented crown-segmentation framework for UAV RGB imagery. The proposed model is built on the U-Net architecture, where LSNet [22] serves as the encoder to strengthen multi-scale feature extraction, while the HPA module [23] is introduced into the skip connections to achieve attention-enhanced multi-scale feature fusion. Through this design, the model improves contextual feature interaction and boundary-sensitive representation, providing a more effective solution for segmenting *Haloxylon ammodendron* crowns under complex desert conditions.

## Materials and methods

### Overview of the study area

The Junggar Basin, located in northern Xinjiang, extends from 42°36’18”N to 48°39’30”N and from 82°17’44”E to 96°01’13”E. Bordered by the Altai Mountains to the northeast, the Tianshan Mountains to the south, and the Western Junggar Mountains to the west, the basin has a triangular structure. It is the second-largest inland basin in China, covering an area of approximately 380,000 km^2^, with elevations ranging from 189 to 1569 m. The terrain features higher elevations in the east and lower elevations in the west [35].

At the center of the basin lies the Gurbantunggut Desert, the largest fixed and semi-fixed desert in China. Due to its unique topography, the climate in the basin varies significantly, with an annual mean temperature ranging from 2 to 11 ° C, annual precipitation between 20 and 295 mm, and annual rainy days from 14 to 107 (defined as days with daily precipitation ≥0.1 mm). The annual mean relative humidity fluctuates between 33% and 62%, and the annual sunshine duration spans from 2542 to 3335 h [36].

Vegetation in the basin is sparsely distributed, with dominant species including *Haloxylon ammodendron*, *Haloxylon persicum*, *Reaumuria soongorica*, *Tamarix*, *Halostachys caspica*, and early-spring ephemeral plants. The predominant soil types are brown calcic soil and desert gray-calcic soil, with specialized types such as takyr soil, meadow soil, and saline-alkali soil occurring locally [37].

**Fig 1:**
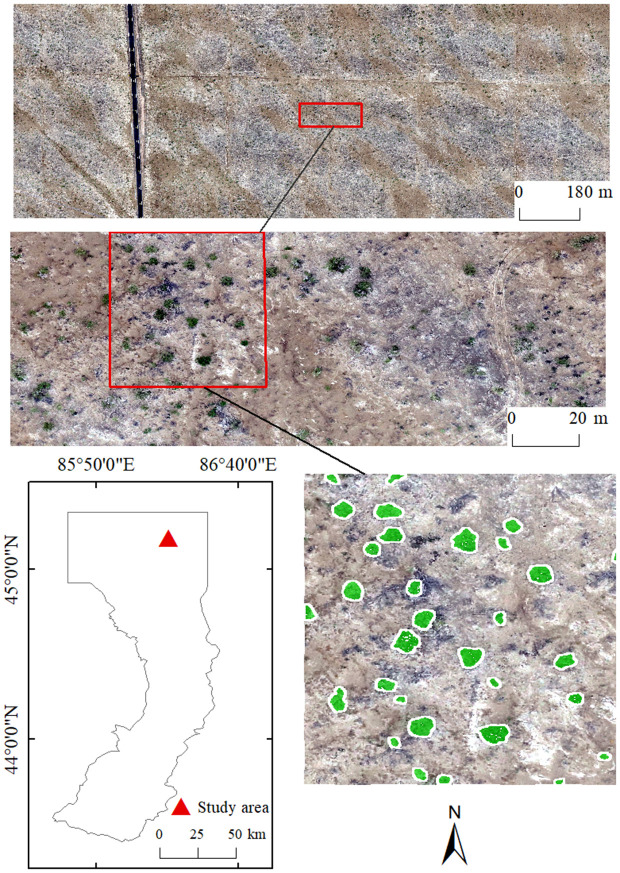
Location of the study area and examples of UAV RGB imagery.

Fig 1 shows the location of the study area and examples of UAV imagery.

### Data sources and dataset preparation

In this study, high-resolution UAV RGB orthomosaic imagery covering natural *Haloxylon ammodendron* stands in the Junggar Basin was used for crown segmentation analysis. The imagery was acquired in July 2023 using a DJI Matrice 300 RTK (M300) UAV equipped with a Zenmuse L1 sensor. Flight missions were conducted along pre-programmed routes, and the collected RGB images were processed using DJI Terra to generate a large orthomosaic of the study area.

For dataset construction, the orthomosaic was cropped into image patches of 640 × 640 pixels, resulting in a total of 1600 RGB image patches. The dataset was divided into training, validation, and test sets at a ratio of 70%, 15%, and 15%, respectively. To prevent data leakage, the three subsets were assigned from different flight lines, ensuring that adjacent image patches did not appear in multiple subsets. This strategy minimized spatial dependence among subsets, providing a more reliable evaluation of the model’s generalization performance.

All image patches were manually annotated for crown segmentation. During dataset preparation, only one training image patch without visible *Haloxylon ammodendron* crowns was retained, while all validation and test patches contained foreground crowns. Across the entire dataset, foreground crown pixels accounted for 4.71% of all valid pixels, with background pixels accounting for 95.29%, resulting in a background-to-foreground ratio of approximately 20.25:1. This distribution highlights a clear pixel-level class imbalance, which should be considered when interpreting the segmentation results.

The resulting dataset was used for model training, validation, and independent test evaluation.

### Methods

#### Introduction to the U-Net network model.

**Fig 2:**
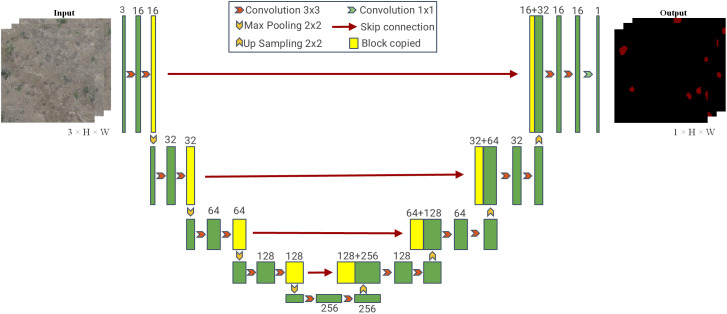
U-Net network structure.

U-Net [13] is a widely used semantic segmentation network featuring a symmetric encoder-decoder architecture, consisting of a contracting path for feature extraction and an expansive path for spatial recovery (Fig 2). Through skip connections, U-Net effectively combines deep semantic information with shallow spatial details, making it well-suited for pixel-level prediction tasks in remote sensing imagery.

In this study, U-Net was used as the baseline segmentation framework. Its encoder-decoder architecture and skip-connection design serve as the structural foundation for the proposed model, and its performance provides an important benchmark for evaluating the effectiveness of the subsequent improvements.

During training, Dice loss was employed to optimize segmentation performance, as defined in Eqs. (1) and (2):


$$\begin{array}{c} Dice=\frac{2\sum_{i}p_{i}g_{i}+\varepsilon }{\sum_{i}p_{i}+\sum_{i}g_{i}+\varepsilon } \end{array}$$ (1)

$$\begin{array}{c} {Loss}_{Dice}=1-Dice \end{array}$$ (2)

where *p* and *g* denote the prediction and the ground truth, respectively, and ε is a small constant introduced for numerical stability.

#### Introduction to the HPA-UNet-LSNet neural network.

**Fig 3:**
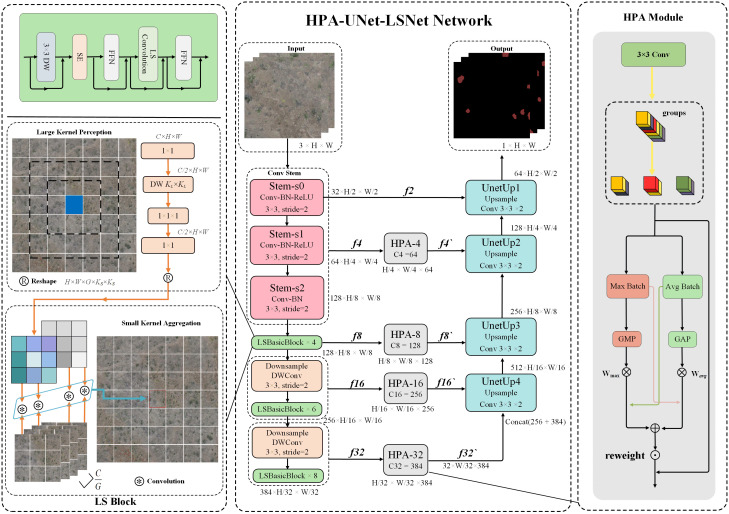
HPA-UNet-LSNet network structure.

Although U-Net has shown strong performance as a general semantic segmentation framework, further adaptation is necessary for the specific task of *Haloxylon ammodendron* crown segmentation from UAV RGB imagery. The main challenges arise from large variations in crown size, complex sandy backgrounds, and the similarity in color and texture between crowns and surrounding soil or co-occurring shrubs. These factors complicate accurate crown delineation, especially for small individuals and crowns with blurred boundaries. To address these issues, we propose HPA-UNet-LSNet, an enhanced U-Net-based segmentation model. Its overall architecture is shown in Fig 3.

The main modifications of the proposed architecture are summarized as follows:

1. The original downsampling path of U-Net is replaced with an LSNet encoder [22]. By combining large-kernel perception with small-kernel aggregation, LSNet enables efficient multi-scale feature extraction, improving the representation of both global context and local crown details. This is particularly beneficial for distinguishing *Haloxylon ammodendron* crowns from sandy backgrounds and other shrubs in UAV RGB imagery.
2. The HPA module [23] is integrated along the skip connection pathway between the encoder and decoder. Through hybrid pooling-based attention aggregation, HPA enhances structural representation and boundary-sensitive feature fusion, allowing the network to better preserve crown contours and improve the discrimination between canopy regions and background areas.
3. The U-shaped encoder-decoder structure of U-Net is retained to ensure the progressive recovery of spatial resolution. With the collaboration of LSNet-based downsampling and HPA-enhanced skip connection fusion, the decoder can more effectively integrate deep semantic features with high-resolution spatial details, thereby improving the pixel-level segmentation accuracy of *Haloxylon ammodendron* crowns in UAV imagery.

LSNet is particularly suitable as the downsampling encoder because it can efficiently capture both broad contextual information and fine-scale local structures. For *Haloxylon ammodendron* crown segmentation, this is crucial because crown shapes vary significantly across individuals, while the surrounding sandy background often introduces strong visual interference. By enhancing multi-scale feature extraction, LSNet improves the model’s ability to distinguish crown patterns from background textures and increases robustness across different crown sizes.

The HPA module is introduced along the skip connection pathway to further improve feature fusion between the encoder and decoder. Its hybrid pooling mechanism enhances attention to structurally informative regions and strengthens boundary-sensitive responses, which is especially useful for crowns with irregular shapes and weak edges. As a result, the proposed HPA-UNet-LSNet can better preserve fine crown structures while suppressing background noise, providing stronger support for accurate segmentation of *Haloxylon ammodendron* crowns from UAV RGB imagery.

#### Permissions and fieldwork compliance.

The UAV data used in this study were collected in non-protected areas within the Junggar Basin, Xinjiang, China. All fieldwork and UAV operations complied with applicable local regulations. The data acquisition did not involve protected areas or endangered species and did not cause ecological disturbance.

### Accuracy evaluation metrics

To comprehensively evaluate the segmentation performance, both instance-level and pixel-level metrics were employed in this study.

At the instance level, true positives (TP), false positives (FP), and false negatives (FN) were determined by matching predicted crowns with reference crowns. Each predicted crown was matched to at most one reference crown, and each reference crown was matched to at most one predicted crown. A predicted crown was considered a true positive when its Intersection over Union (IoU) with a reference crown was at least 0.5. Otherwise, unmatched predicted crowns and unmatched reference crowns were counted as false positives and false negatives, respectively. Here, TP (true positive) represents the number of *Haloxylon ammodendron* individuals correctly identified, FP (false positive) indicates the number of predicted crown instances that do not correspond to reference *Haloxylon ammodendron* crowns, and FN (false negative) represents the number of reference *Haloxylon ammodendron* individuals that were missed. Based on these instance-level counts, Precision, Recall, and F1-score were calculated as follows:


$$\begin{array}{c} Precision=\frac{TP}{TP+FP} \end{array}$$ (3)

$$\begin{array}{c} Recall=\frac{TP}{TP+FN} \end{array}$$ (4)

$$\begin{array}{c} F1\text{-}Score=\frac{2\times Precision\times Recall}{Precision+Recall} \end{array}$$ (5)

Recall reflects the model’s ability to detect target individuals, Precision indicates the reliability of the predicted individuals, and the F1-score provides a balanced assessment of omission and commission errors at the individual crown level.

At the pixel level, mean Intersection over Union (mIoU) was used to evaluate the spatial overlap between the predicted segmentation map and the ground-truth annotation. For the binary segmentation task, mIoU was computed as the average IoU over the two classes (crown and background):


$$\begin{array}{c} mIoU=\frac{1}{2}\left(\frac{TP_{c}}{TP_{c}+FP_{c}+FN_{c}}+\frac{TP_{b}}{TP_{b}+FP_{b}+FN_{b}}\right) \end{array}$$ (6)

where *TP*_c_, *FP*_c_, and *FN*_c_ represent the pixel-level true positives, false positives, and false negatives for the crown class (denoted by ‘c‘), respectively. Similarly, *TP*_b_, *FP*_b_, and *FN*_b_ correspond to the pixel-level true positives, false positives, and false negatives for the background class (denoted by ‘b‘). Therefore, the mIoU reflects the overall pixel-level segmentation accuracy and spatial overlap quality of the predicted crown masks by averaging the intersection-over-union (IoU) values for both the crown and background classes.

## Results

### Experimental setup

All experiments were conducted on a desktop computer running Windows 11, utilizing the PyTorch framework. The software environment included Python 3.10, CUDA 11.8, and cuDNN 8.5.0, while the hardware platform was equipped with 16 GB RAM and an NVIDIA GeForce RTX 4070 graphics card.

The dataset used in this study consisted of 1600 UAV RGB image patches cropped from the orthomosaic, each with a spatial size of 640 × 640 pixels. These samples were divided into training, validation, and test sets at a ratio of 70%, 15%, and 15%, respectively. To prevent data leakage, the training, validation, and test subsets were assigned from different flight lines, ensuring that spatially adjacent image patches did not appear in multiple subsets. This strategy minimized spatial dependence between subsets, providing a more reliable evaluation of the model’s generalization performance.

At the pixel level, the dataset exhibited a clear class imbalance. Across the entire dataset, foreground crown pixels accounted for 4.71% of all valid pixels, while background pixels accounted for 95.29%, corresponding to a background-to-foreground ratio of approximately 20.25:1. This distribution was taken into account when interpreting the segmentation results.

To mitigate the influence of random initialization, each ablation setting was independently trained using three random seeds (11, 12, and 13). The quantitative results reported in this study are expressed as mean ± standard deviation across the three runs. For qualitative comparison, the checkpoint with validation performance closest to the mean result across the three runs was selected as the representative model, ensuring that the displayed predictions reflected typical rather than selectively optimal performance.

Unless otherwise noted, all quantitative metrics reported in the Results section were computed on the independent test set, with the validation set used solely for model selection and training monitoring. To enhance comparability, all models were evaluated under a unified experimental protocol, which included the same data split, training schedule, and evaluation procedure. The shared training hyperparameter settings used for all compared models are summarized in S1 Appendix.

In addition to reporting averaged results across repeated runs, statistical significance was further assessed at the per-image level on the independent test set. Specifically, paired Wilcoxon signed-rank tests were performed between the baseline U-Net and HPA-UNet-LSNet using per-image pixel-level IoU, Precision, Recall, and F1-score values. The corresponding results are provided in the Appendix.

### Ablation experiment

**Fig 4:**
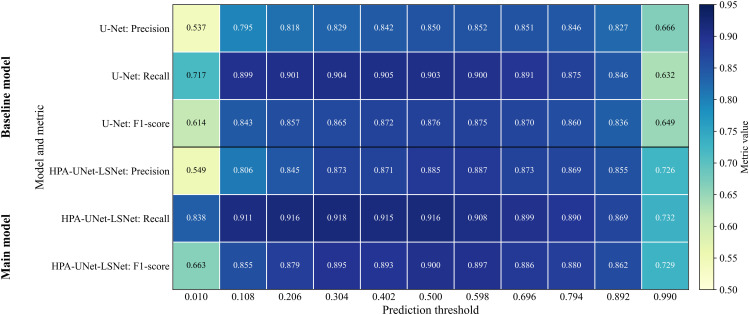
Heatmap of threshold-dependent Precision, Recall, and F1-score for the baseline and main models. The figure summarizes the metric values obtained at different prediction thresholds using the best-weight checkpoint of each model. Overall, HPA-UNet-LSNet maintains higher Precision and F1-score over most thresholds while preserving high Recall.

**Fig 5:**
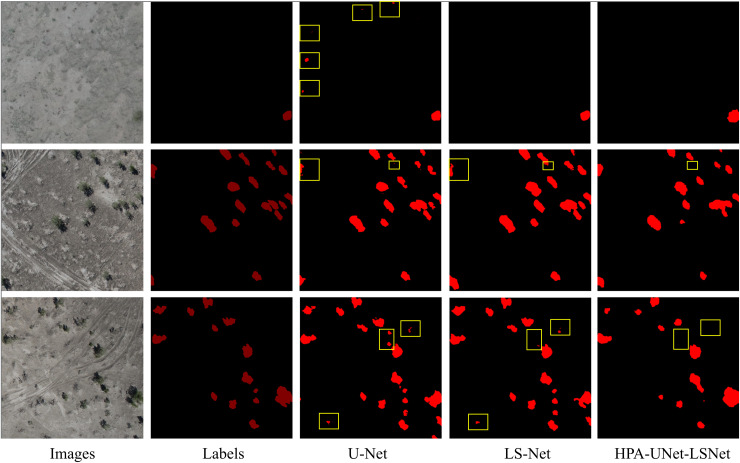
Prediction results of the ablation experiment. From left to right, the columns show the input images, reference labels, baseline U-Net predictions, LS-U-Net predictions, and HPA-UNet-LSNet predictions. The yellow boxes highlight representative differences in small-target detection and background suppression among the models.

To examine the contributions of the LSNet encoder and the HPA module, an ablation study was conducted by progressively introducing these two components into the baseline U-Net. All experiments were performed under the same training environment and hyperparameter settings. For clarity, the U-Net variant in which the original encoder was replaced by LSNet is referred to as **LS-U-Net**. The quantitative results are presented in Table 1, the threshold-dependent metric comparison between the baseline and main models is shown in Fig 4, and representative prediction results are shown in Fig 5.

**Table 1:** Ablation results of different module combinations.

LSNet	HPA	TP	FP	FN	Precision	Recall	F1-score	mIoU
×	×	2096 ± 11	454 ± 53	224 ± 11	0.8222 ± 0.0173	0.9035 ± 0.0046	0.8608 ± 0.0099	0.8222 ± 0.0060
✓	×	2129 ± 8	331 ± 37	191 ± 8	0.8655 ± 0.0132	0.9178 ± 0.0042	0.8908 ± 0.0083	0.8432 ± 0.0044
✓	✓	2134 ± 12	267 ± 18	185 ± 10	0.8890 ± 0.0063	0.9198 ± 0.0051	0.9041 ± 0.0032	0.8456 ± 0.0043

**Note:** ✓ indicates that the corresponding module was used, whereas × indicates that it was not used. TP, FP, and FN denote the numbers of detected *Haloxylon ammodendron* individuals and are reported as mean ± standard deviation over three runs.

As shown in Table 1, segmentation performance improved progressively as LSNet and HPA were introduced into the baseline U-Net. Compared with U-Net, LS-U-Net increased TP from 2096 ± 11 to 2129 ± 8 and reduced FP from 454 ± 53 to 331 ± 37. At the same time, Precision, Recall, F1-score, and mIoU increased from 0.8222 ± 0.0173, 0.9035 ± 0.0046, 0.8608 ± 0.0099, and 0.8222 ± 0.0060 to 0.8655 ± 0.0132, 0.9178 ± 0.0042, 0.8908 ± 0.0083, and 0.8432 ± 0.0044, respectively. These results indicate that replacing the original U-Net encoder with LSNet improved multi-scale feature extraction and reduced confusion with the desert background.

After the HPA module was further introduced, the complete HPA-UNet-LSNet achieved the best overall performance among the ablation settings. TP increased to 2134 ± 12, while FP and FN decreased to 267 ± 18 and 185 ± 10, respectively. Precision, Recall, F1-score, and mIoU further increased to 0.8890 ± 0.0063, 0.9198 ± 0.0051, 0.9041 ± 0.0032, and 0.8456 ± 0.0043, respectively. Relative to the baseline U-Net, this corresponds to improvements of 0.0668 in Precision, 0.0163 in Recall, 0.0433 in F1-score, and 0.0234 in mIoU, together with average reductions of approximately 187 false positives and 39 false negatives. These results suggest that HPA provided additional benefit beyond LS-U-Net, especially in suppressing false detections while preserving high detection completeness.

To further examine the Precision–Recall trade-off, Fig 4 summarizes the threshold-dependent Precision, Recall, and F1-score values of the baseline U-Net and HPA-UNet-LSNet. The heatmap was computed from the best-weight checkpoint of each model. Across most thresholds, the main model maintained higher Precision and F1-score than the baseline model, while Recall also remained slightly higher within the main operating range. Around the commonly used threshold of 0.5, the baseline model achieved Precision, Recall, and F1-score values of 0.850, 0.903, and 0.876, respectively, whereas the main model achieved 0.885, 0.916, and 0.900. This indicates that the improvement was not limited to a single operating point, but remained stable across a range of thresholds. At present, no established ecological survey guideline or expert-consensus threshold is available for defining acceptable false-positive and false-negative rates in *Haloxylon ammodendron* crown monitoring. Therefore, the threshold of 0.5 was treated as a neutral default operating point. This choice is also consistent with widely used evaluation practice in object detection and instance segmentation, where an IoU threshold of 0.5 has long been adopted as a basic criterion for a correct match in the PASCAL VOC protocol, is explicitly reflected in COCO-style AP50 reporting, and remains a commonly used reference threshold in detector design and evaluation [11,38–40]. Under this threshold, HPA-UNet-LSNet preserved high Recall while achieving higher Precision and F1-score than the baseline model, indicating a more favorable trade-off between ecologically relevant error types.

To assess whether the improvement over the baseline U-Net was statistically significant, a paired Wilcoxon signed-rank test was conducted on per-image results from the test set (S1 Appendix). The results showed that HPA-UNet-LSNet achieved significantly higher IoU, Precision, Recall, and F1-score than the baseline U-Net (*p* < 0.001 for all metrics). This indicates that the observed improvement was not only reflected in the aggregated results, but was also statistically significant at the per-image level.

The visual results in Fig 5 are consistent with the quantitative findings. The baseline U-Net still shows noticeable false detections and incomplete segmentation in some samples, particularly for small crowns and weak-boundary regions. After LS-U-Net was introduced, the predicted masks became cleaner and more consistent with the reference labels, although local omissions remained. With the further addition of HPA, the predicted crown regions show better agreement with the labels, with fewer false positives and more complete crown delineation. The yellow boxes highlight representative differences in small-target detection and background suppression across the models.

Overall, the ablation results indicate that both LSNet and HPA contributed positively to *Haloxylon ammodendron* crown segmentation. LSNet mainly improved the encoder representation, whereas HPA further refined feature fusion and foreground–background discrimination. Their combination produced the best performance among the tested ablation settings.

### Comparative analysis of accuracy among different semantic segmentation models

**Fig 6:**
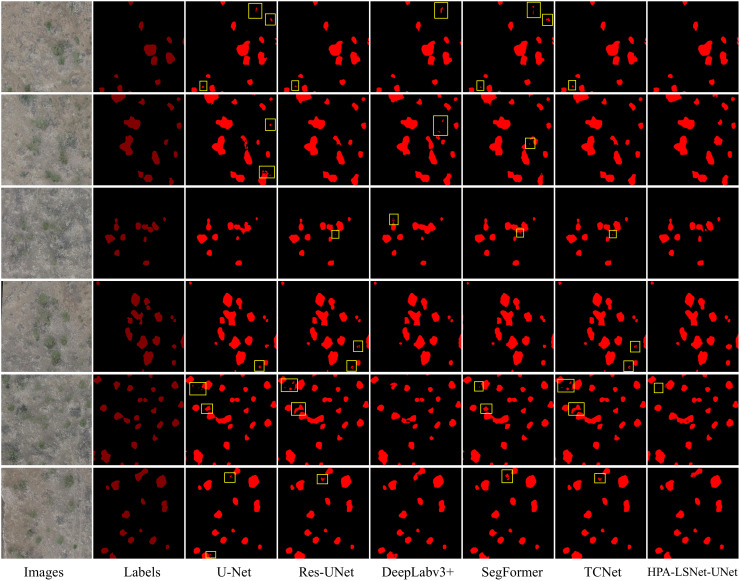
Prediction results of comparative experiments.

To compare segmentation performance across models, experiments were conducted using U-Net, Res-U-Net, DeepLabv3 + , SegFormer, TCNet, and the proposed HPA-UNet-LSNet. The quantitative results are summarized in Table 2, and representative prediction results are shown in Fig 6.

**Table 2:** Comparison of model accuracies.

Model	TP	FP	FN	Precision	Recall	F1-score	mIoU
U-Net	2096 ± 11	454 ± 53	224 ± 11	0.8222 ± 0.0173	0.9035 ± 0.0046	0.8608 ± 0.0099	0.8222 ± 0.0060
Res-U-Net	2121 ± 25	420 ± 27	199 ± 25	0.8346 ± 0.0086	0.9141 ± 0.0108	0.8725 ± 0.0067	0.8334 ± 0.0141
DeepLabv3+	2082 ± 18	437 ± 24	238 ± 18	0.8267 ± 0.0069	0.8974 ± 0.0076	0.8606 ± 0.0032	0.8416 ± 0.0012
SegFormer	2055 ± 7	374 ± 28	265 ± 7	0.8460 ± 0.0094	0.8856 ± 0.0029	0.8653 ± 0.0038	0.8242 ± 0.0015
TCNet	2105 ± 18	281 ± 22	215 ± 18	0.8823 ± 0.0071	0.9072 ± 0.0077	0.8945 ± 0.0009	**0.8536 ± 0.0002**
HPA-UNet-LSNet	**2134 ± 12**	**267 ± 18**	**185 ± 10**	**0.8890 ± 0.0063**	**0.9198 ± 0.0051**	**0.9041 ± 0.0032**	0.8456 ± 0.0043

*Note:* TP, FP, and FN denote the numbers of detected *Haloxylon ammodendron* individuals and are reported as rounded mean ± standard deviation over three runs. Precision, Recall, F1-score, and mIoU are reported as mean ± standard deviation over three runs.

As shown in Table 2, HPA-UNet-LSNet achieved the best instance-level performance, with the highest Precision (0.8890), Recall (0.9198), and F1-score (0.9041), as well as the highest TP and the lowest FN. These results indicate that the proposed model is more effective in identifying crown targets while reducing missed detections.

TCNet achieved the highest mIoU (0.8536), slightly higher than that of HPA-UNet-LSNet (0.8456). This suggests that TCNet had a slight advantage in pixel-level overlap, whereas HPA-UNet-LSNet demonstrated stronger balance between precision and recall at the instance level. For ecological crown monitoring, this balance is important because omission and commission errors directly affect the estimation of crown abundance and spatial distribution.

Res-U-Net and DeepLabv3 + showed intermediate performance. Res-U-Net achieved an F1-score of 0.8725 and an mIoU of 0.8334, while DeepLabv3 + achieved a similar F1-score (0.8606) and a slightly higher mIoU (0.8416). SegFormer achieved relatively high Precision (0.8460), but its Recall and F1-score were lower than those of TCNet and HPA-UNet-LSNet. U-Net exhibited the weakest overall performance, with the lowest Precision and relatively high false positives, indicating limited discrimination between crowns and the complex desert background.

Overall, HPA-UNet-LSNet demonstrated the most competitive performance for *Haloxylon ammodendron* crown segmentation. Although TCNet achieved the highest mIoU, HPA-UNet-LSNet outperformed the other models in Precision, Recall, and F1-score, indicating stronger robustness for instance-level crown extraction in complex desert environments.

Fig 6 shows representative prediction results for the models compared. U-Net produced more redundant regions and false detections. Res-U-Net improved crown completeness, but inaccurate boundaries remained in some samples. DeepLabv3 + generated relatively stable pixel-level masks, though some crowns were still fragmented. SegFormer reduced false positives to some extent, but missed detections persisted in sparse crown areas. TCNet produced cleaner and more compact masks, consistent with its strong pixel-level performance.

By contrast, HPA-UNet-LSNet generated more balanced results, with fewer false detections and more complete crown delineation. Its advantages were most evident for sparse, small, and irregular crowns. These visual results are consistent with the quantitative comparison and further support the effectiveness of the proposed model for crown segmentation in complex desert environments.

### Qualitative analysis of *Haloxylon ammodendron* crown segmentation using grad-CAM heatmaps

To further analyze the feature-response characteristics of HPA-UNet-LSNet in *Haloxylon ammodendron* crown segmentation, Grad-CAM was employed to visualize the activation patterns of both the baseline model and the proposed model from foreground and background perspectives.

The *Up Concat1 Conv2* layer was selected for observation as it is located in the late decoding stage, where low-level spatial details and high-level semantic information are integrated. The responses at this layer thus provide an intuitive view of how the model focuses on crown regions and suppresses background interference before the final prediction.

**Fig 7:**
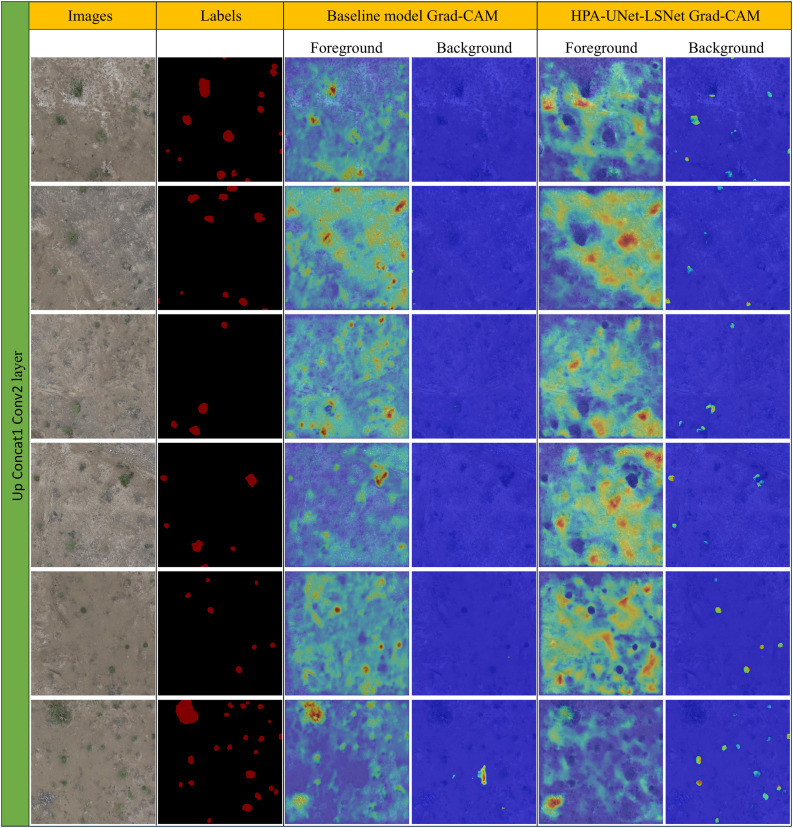
Grad-CAM comparison between the baseline model and HPA-UNet-LSNet at the Up Concat1 Conv2 layer. Compared to the baseline model, HPA-UNet-LSNet shows more concentrated foreground responses on crown regions and reduced activation in irrelevant background areas.

As shown in Fig 7, the foreground Grad-CAM results of the baseline model are relatively scattered, with activated regions not always well-constrained to the crown areas. In several instances, responses extend into surrounding background regions, indicating limited discrimination between target crowns and non-target areas. In contrast, the foreground responses of HPA-UNet-LSNet are generally more concentrated and better aligned with the labeled crown locations. This suggests that the proposed model is more effective in capturing crown-related features during the decoding stage.

The background Grad-CAM results exhibit a similar trend. For the baseline model, non-target regions still show noticeable activations in some samples, suggesting that background responses are not fully suppressed. In contrast, HPA-UNet-LSNet produces more localized background activations, while large irrelevant areas remain relatively inactive. At the same time, crown regions are more distinctly separated from the surrounding background response pattern. This indicates that the proposed model provides a more distinct foreground-background representation under complex desert conditions.

Overall, the Grad-CAM comparison indicates that HPA-UNet-LSNet generates more focused responses on crown regions while reducing background interference. These qualitative observations align with the quantitative evaluation results and provide additional evidence that the proposed model improves feature discrimination for *Haloxylon ammodendron* crown extraction.

**Fig 8:**
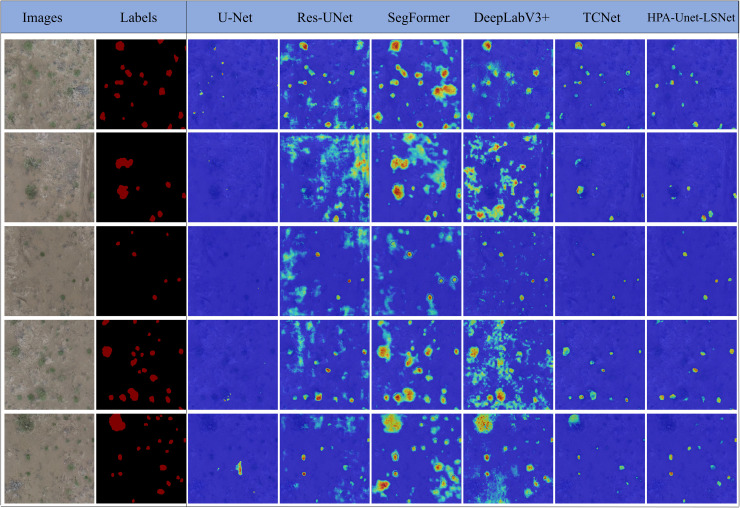
Comparison of Grad-CAM heatmaps among different models for *Haloxylon ammodendron* crown segmentation. The figure shows representative heatmap responses of U-Net, Res-U-Net, SegFormer, DeepLabv3 + , TCNet, and HPA-UNet-LSNet. In the illustrated samples, HPA-UNet-LSNet tends to produce relatively concentrated responses on crown regions and comparatively limited activation in surrounding background areas.

To further compare feature-response patterns among different segmentation models, Grad-CAM heatmaps were visualized for representative samples, as shown in Fig 8. These heatmaps provide complementary qualitative evidence to interpret the quantitative differences reported in Table 2.

From the perspective of crown-related responses, the compared models show clear differences in activation distribution. U-Net generally exhibits sparse or weak responses in some crown regions, which may be associated with incomplete coverage of small or low-contrast crowns. Res-U-Net improves response continuity to some extent, although activations remain dispersed in several patches. SegFormer and DeepLabv3 + produce broader response regions in many cases, indicating that they can capture crown-related information, but the activated areas are sometimes less compact and may extend into nearby non-target regions. TCNet shows relatively concentrated responses in several samples, reflecting comparatively stable attention to crown structures. HPA-UNet-LSNet also presents concentrated crown-related activations in most illustrated cases, with its high-response regions generally more consistent with the labeled targets.

From the perspective of background responses, differences among the models are also apparent. In U-Net and several other models, scattered activations remain visible in non-crown areas, suggesting that background interference is not completely suppressed. By contrast, TCNet and HPA-UNet-LSNet tend to show cleaner background response distributions in many patches, with fewer high-activation regions outside the labeled crowns. This pattern implies a clearer separation between crown targets and the surrounding desert background.

In general, the Grad-CAM results are consistent with the quantitative comparison. Although all models respond to crown regions to some extent, HPA-UNet-LSNet tends to show more concentrated crown-related activations and comparatively restrained background responses in the illustrated examples. These qualitative results further support the effectiveness of the proposed architecture for *Haloxylon ammodendron* crown segmentation in complex desert environments.

## Discussion

### Supplementary analysis of scale-specific performance and model complexity

To complement the overall quantitative results, this study further examined the scale-specific segmentation performance of HPA-UNet-LSNet and compared its model complexity with that of representative benchmark models. This analysis provides valuable insight into two aspects: (1) how the model performs on *Haloxylon ammodendron* crowns of different sizes, and (2) whether the achieved accuracy comes with an acceptable storage and parameter burden.

Table 3 summarizes the size-stratified evaluation results of U-Net and HPA-UNet-LSNet. Based on the ground-truth crown area, all targets were categorized into three bins: small, medium, and large crowns. The values are reported as mean ± standard deviation over three runs. As shown in the table, the performance difference between the two models is most apparent in the small-target group. For small crowns, HPA-UNet-LSNet achieved higher TP (675 ± 15 vs. 667 ± 18), lower FP (338 ± 2 vs. 396 ± 41), and fewer FN (85 ± 15 vs. 93 ± 18) than U-Net. The corresponding Precision, Recall, F1-score, and mIoU increased from 0.6282 ± 0.0240, 0.8772 ± 0.0231, 0.7318 ± 0.0179, and 0.6498 ± 0.0045 to 0.6662 ± 0.0043, 0.8877 ± 0.0201, 0.7611 ± 0.0102, and 0.6929 ± 0.0089, respectively. These results indicate that the proposed model provides more reliable feature representation for weak, sparse, and small crown targets, which are typically the most challenging cases in desert UAV imagery.

**Table 3:** Size-stratified segmentation results of U-Net and HPA-UNet-LSNet.

Model	Size bin	TP	FP	FN	Precision	Recall	F1-score	mIoU
U-Net	Small	667 ± 18	396 ± 41	93 ± 18	0.6282 ± 0.0240	0.8772 ± 0.0231	0.7318 ± 0.0179	0.6498 ± 0.0045
U-Net	Medium	704 ± 5	31 ± 7	54 ± 5	0.9579 ± 0.0092	0.9288 ± 0.0060	0.9431 ± 0.0027	0.7688 ± 0.0100
U-Net	Large	741 ± 2	35 ± 3	41 ± 2	0.9545 ± 0.0032	0.9475 ± 0.0022	0.9510 ± 0.0027	0.8403 ± 0.0073
HPA-UNet-LSNet	Small	675 ± 15	338 ± 2	85 ± 15	0.6662 ± 0.0043	0.8877 ± 0.0201	0.7611 ± 0.0102	0.6929 ± 0.0089
HPA-UNet-LSNet	Medium	704 ± 7	23 ± 9	54 ± 7	0.9685 ± 0.0115	0.9283 ± 0.0088	0.9479 ± 0.0010	0.7893 ± 0.0053
HPA-UNet-LSNet	Large	751 ± 2	31 ± 3	31 ± 2	0.9604 ± 0.0032	0.9599 ± 0.0019	0.9601 ± 0.0007	0.8561 ± 0.0045

**Note:** Targets were stratified into small, medium, and large bins according to ground-truth crown area. TP, FP, and FN denote instance-level counts, and all values are reported as mean ± standard deviation over three runs.

For medium-sized crowns, the two models showed very similar TP and FN values overall, but HPA-UNet-LSNet still reduced FP from 31 ± 7 to 23 ± 9 and improved Precision from 0.9579 ± 0.0092 to 0.9685 ± 0.0115. Its F1-score and mIoU also increased slightly, from 0.9431 ± 0.0027 and 0.7688 ± 0.0100 to 0.9479 ± 0.0010 and 0.7893 ± 0.0053, respectively. For large crowns, both models achieved relatively high segmentation accuracy overall, but HPA-UNet-LSNet still maintained a slight advantage, with higher TP, lower FN, and better overlap quality. Specifically, its mIoU for large crowns reached 0.8561 ± 0.0045, compared with 0.8403 ± 0.0073 for U-Net.

Overall, the size-stratified analysis indicates that the improvement brought by HPA-UNet-LSNet is not limited to the aggregated metrics. The gain is most evident for small crowns, while moderate but consistent improvements are observed for medium and large crowns. This pattern is significant for practical monitoring, as missing small and sparsely distributed individuals can directly affect the interpretation of crown density, regeneration status, and population structure in desert ecosystems.

**Fig 9:**
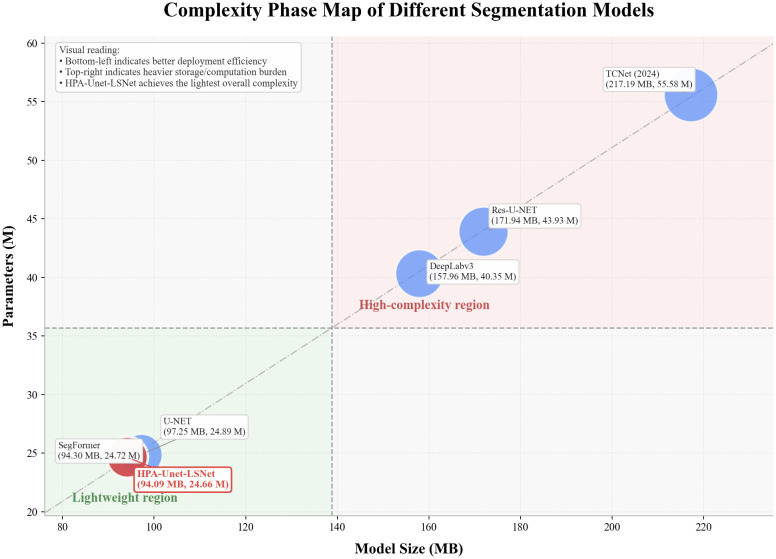
Complexity phase map of different segmentation models. The horizontal axis denotes model size, and the vertical axis denotes the number of parameters. Models located closer to the lower-left corner generally indicate lower storage and parameter burden. For deployment reference, the average inference speeds for a 640 × 640 image patch on the RTX 4070 were 25.11, 44.59, 74.97, 33.25, 93.14, and 47.73 images/s for U-Net, Res-U-Net, DeepLabv3 + , SegFormer, TCNet, and HPA-UNet-LSNet, respectively. HPA-UNet-LSNet is positioned in the lightweight region while maintaining competitive segmentation performance and a moderate inference speed.

In addition to segmentation accuracy, model complexity is an important consideration for UAV-based ecological monitoring, especially when models are expected to be deployed in resource-constrained environments or used for large-scale inference [41,42]. Fig 9 presents the complexity phase map of different segmentation models, where the horizontal axis represents model size and the vertical axis represents the number of parameters. Models positioned closer to the lower-left corner generally require less storage and involve a lower computational burden, making them more deployment-friendly.

As shown in Fig 9, DeepLabv3 + , Res-U-Net, and TCNet are located in the upper-right region, indicating relatively high complexity in both storage and parameter scale. U-Net and SegFormer are positioned in the lower-left region, reflecting more lightweight configurations. HPA-UNet-LSNet is also positioned in this lightweight region, slightly lower than U-Net and SegFormer in both model size and parameter count, with a model size of 94.09 MB and 24.66 M parameters. This suggests that the proposed model maintains a compact structure while achieving competitive segmentation performance in the previous experiments.

For deployment reference, the average inference speeds on the RTX 4070 for a 640 × 640 image patch were 25.11, 44.59, 74.97, 33.25, 93.14, and 47.73 images/s for U-Net, Res-U-Net, DeepLabv3 + , SegFormer, TCNet, and HPA-UNet-LSNet, respectively, corresponding to approximately 39.82, 22.43, 13.34, 30.08, 10.74, and 20.95 ms per patch. These results indicate that the inference speed of HPA-UNet-LSNet is moderate among the compared models: it is slower than TCNet and DeepLabv3 + , but faster than U-Net, Res-U-Net, and SegFormer. Therefore, although the proposed model is not the fastest in absolute terms, its runtime remains compatible with practical patch-based UAV image inference.

When the complexity map is interpreted together with the quantitative results, HPA-UNet-LSNet demonstrates a favorable balance between efficiency and accuracy. Compared to heavier models such as Res-U-Net, DeepLabv3 + , and TCNet, it requires less storage and fewer parameters. At the same time, compared to the lightweight baseline U-Net, it provides better overall segmentation performance, particularly for small crowns. Together with its moderate inference speed, these results suggest that the proposed model achieves its improvement not through a substantial increase in deployment burden, but through a more effective combination of encoder representation and feature fusion design.

Taken together, the scale-specific results and the complexity analysis provide additional support for the practical value of HPA-UNet-LSNet. The model shows improved segmentation capability for crowns of different sizes, especially small targets, while retaining a relatively lightweight configuration and a moderate inference speed. This characteristic makes it suitable not only for improving segmentation accuracy in research settings but also for potential application in UAV-based vegetation monitoring tasks where both accuracy and computational efficiency are critical.

### Academic dialogue with existing research and innovation positioning

Existing studies on vegetation and tree-crown segmentation have primarily focused on specific species, dense-canopy plantations, orchards, or urban forests, with methodologies that differ significantly from the requirements of *Haloxylon ammodendron* crown segmentation in desert environments. In UAV RGB imagery of arid regions, the target crowns are typically sparse, irregular in shape, and often surrounded by spectrally similar sandy backgrounds and co-occurring shrubs. These characteristics place greater demands on multi-scale feature extraction, foreground-background discrimination, and boundary-sensitive representation.

From the perspective of attention-based modeling, Huang et al. [43] proposed AMDNet, which combines spatial and channel attention through a dual-attention residual design, achieving strong performance in dense tree-species segmentation. Li et al. [44] developed ACE R-CNN, which integrates RGB and canopy-height information to improve instance recognition by combining attention complementation with boundary optimization. These studies demonstrate the value of attention mechanisms for vegetation interpretation. However, their application scenarios differ from the current task. AMDNet was developed primarily for denser vegetation conditions, while ACE R-CNN relies on auxiliary LiDAR-derived information. By contrast, the present study focuses on sparse shrub crowns in desert environments and aims to achieve reliable segmentation using only UAV RGB imagery.

In the realm of semantic segmentation, Ye et al. [45] utilized U^2^-Net for olive crown extraction, achieving high segmentation accuracy under dense-canopy orchard conditions. Duan et al. [46] proposed CIA-UNet for tree-crown segmentation and demonstrated competitive performance on plantation-style datasets. While these models provide useful references for crown delineation, their target objects typically exhibit stronger canopy continuity, clearer structural organization, and more regular spatial arrangement compared to *Haloxylon ammodendron* in desert areas. As a result, their design priorities do not fully address the sparse distribution, weak boundary contrast, and frequent small-target omission encountered in the present study area.

The proposed HPA-UNet-LSNet is positioned as a targeted extension of the U-Net framework for UAV RGB crown segmentation in arid environments. Its main contribution lies not in introducing a completely new segmentation paradigm, but in adapting the encoder-decoder structure to the characteristics of sparse desert shrub crowns. Specifically, LSNet [22] replaces the original U-Net encoder, enabling more effective extraction of both broad contextual cues and fine local structures through the combination of large-kernel perception and small-kernel aggregation. Additionally, the HPA module [23] is introduced along the feature-fusion pathway to strengthen structural response and improve the integration of crown-related information with boundary-sensitive details. This combination is designed to enhance the separability between *Haloxylon ammodendron* crowns and complex background regions while preserving the advantages of the U-shaped architecture in spatial-detail recovery.

The experimental results demonstrate the effectiveness of the proposed design for the present task. As shown in Table 2, HPA-UNet-LSNet achieved the highest Precision (0.8890), Recall (0.9198), and F1-score (0.9041), as well as the highest mean TP and the lowest mean FN among the compared models. Although TCNet obtained a slightly higher mIoU (0.8536 vs. 0.8456), HPA-UNet-LSNet exhibited a more favorable balance between false positives and false negatives at the instance level.

From a methodological perspective, the contribution of this study is primarily application-oriented. Specifically, LSNet was integrated into the U-Net framework to strengthen multi-scale feature extraction, and the HPA module was incorporated to improve structurally informative feature fusion under sparse-target conditions. Furthermore, the proposed framework provides an RGB-only solution for crown segmentation in desert environments, which is practical for low-cost and large-area monitoring.

Overall, the contribution of this study lies in adapting an encoder-decoder segmentation framework to the specific characteristics of sparse *Haloxylon ammodendron* crowns in complex desert backgrounds. Rather than proposing a new general segmentation paradigm, this study provides a targeted and effective solution for UAV RGB crown segmentation in arid regions.

### Model limitations and analysis of missed detection causes

Although HPA-UNet-LSNet achieved favorable overall performance for *Haloxylon ammodendron* crown segmentation, missed detections still occurred in some test samples. Based on the quantitative results and visual inspection of the predicted masks, these omissions appear to arise primarily from the combined influence of target characteristics, background complexity, and the representational limits of the model.

One of the main reasons for missed detections is the weak visual separability of some *Haloxylon ammodendron* individuals in UAV RGB imagery. In particular, small crowns, sparse seedlings, and partially degraded individuals often occupy only a limited number of pixels. Their spectral and textural differences from the surrounding sandy soil are relatively subtle, making the foreground signal weak. Under such conditions, these targets are more likely to be confused with background regions. Additionally, irregular crown morphology and discontinuous branch structures can reduce the completeness of crown responses, especially when the crown edge is blurred or locally fragmented.

Another important factor is the complexity of the desert background. Co-occurring shrubs, dry vegetation residues, and heterogeneous bare-soil textures may generate local patterns similar to those of *Haloxylon ammodendron* crowns. Although the proposed model improves background suppression compared to the baseline U-Net, these background variations still increase the difficulty of identifying small or weak crown targets. In some cases, when the model focuses on suppressing ambiguous background responses, subtle crown features may also be suppressed, leading to missed detections.

From the model perspective, the remaining missed detections suggest that the current feature representation is still less sensitive to extremely small or weak targets compared to medium- and large-sized crowns. This interpretation is consistent with the size-stratified results. As shown in Table 3, the performance gap between HPA-UNet-LSNet and U-Net is most evident in the small-target group, but the small-crown bin still shows the lowest Precision, F1-score, and mIoU among the three scale categories. This indicates that, although the LSNet encoder and HPA module improve the detection of small crowns, the segmentation of weak and sparse individuals remains the most challenging aspect of the task.

In essence, the remaining missed detections reflect a practical trade-off between target sensitivity and background suppression in complex desert scenes. Improving sensitivity to subtle crown features without substantially increasing false positives remains a key issue for further optimization. Future work may focus on strengthening small-target representation, enhancing boundary-sensitive learning, and expanding samples of extreme cases such as sparse seedlings, degraded crowns, and heavily confused background regions. These improvements may help further reduce omissions while maintaining the current balance between precision and recall.

### Research limitations and future directions

Although HPA-UNet-LSNet achieved favorable performance in *Haloxylon ammodendron* crown segmentation, several limitations remain.

First, while this study incorporated a relatively lightweight encoder design, the degree of lightweight optimization is still limited. As shown in Fig 9, HPA-UNet-LSNet has 24.66 M parameters and a model size of 94.09 MB. Compared with TCNet, Res-U-Net, and DeepLabv3 + , its parameter count is reduced by 55.6%, 43.9%, and 38.9%, respectively. However, its complexity remains close to those of U-Net and SegFormer, indicating that further improvements are possible in deployment efficiency and computational cost [41,47].

Second, the data modality is limited. This study used only UAV RGB imagery and did not incorporate LiDAR point clouds, hyperspectral imagery, or other auxiliary data sources. As a result, the current framework primarily relies on two-dimensional spectral and texture information, while crown structural traits and physiological conditions are not explicitly represented [48–50].

Third, the dataset coverage is still insufficient. The current samples do not fully account for more diverse acquisition conditions, such as stronger wind-sand disturbance, seasonal phenological variation, or more complex mixed-background scenes. Therefore, the generalization ability of the model under broader desert conditions still requires further verification [51].

Future work may focus on three key aspects. First, further lightweight optimization can be achieved through more efficient backbones or model compression strategies, such as pruning, quantization, or distillation [41,47]. Second, multi-source data fusion, particularly the integration of LiDAR and hyperspectral data, could enhance the representation of crown structure and vitality [48–50]. Third, expanding the dataset across seasons, regions, and environmental conditions would help strengthen robustness and transferability [51]. Additionally, extending the framework to include joint tasks such as crown segmentation, health assessment, and biomass estimation may further improve its practical value for desert ecosystem monitoring [52].

## Conclusion

To enhance the segmentation of *Haloxylon ammodendron* crowns from UAV RGB imagery in desert environments, this study developed an improved U-Net-based model, **HPA-UNet-LSNet**, by replacing the original encoder with LSNet and incorporating the HPA module for feature fusion. Based on the experimental results, the main conclusions are as follows:

1. **The combination of LSNet and HPA improved the overall performance of the U-Net framework for desert shrub-crown segmentation.** The ablation results demonstrated that progressively introducing LSNet and HPA consistently enhanced segmentation performance. Compared with the baseline U-Net, the complete HPA-UNet-LSNet increased TP from 2096 ± 11 to 2134 ± 12, while reducing FP from 454 ± 53 to 267 ± 18 and FN from 224 ± 11 to 185 ± 10. Concurrently, Precision, Recall, F1-score, and mIoU increased from 0.8222 ± 0.0173, 0.9035 ± 0.0046, 0.8608 ± 0.0099, and 0.8222 ± 0.0060 to 0.8890 ± 0.0063, 0.9198 ± 0.0051, 0.9041 ± 0.0032, and 0.8456 ± 0.0043, respectively. These results highlight the effectiveness of the proposed architectural combination in improving crown segmentation under complex desert backgrounds.
2. **HPA-UNet-LSNet demonstrated competitive performance among the compared models, especially in instance-level accuracy and small-target segmentation.** Among the compared models, HPA-UNet-LSNet achieved the highest mean Precision (0.8890), Recall (0.9198), and F1-score (0.9041), along with the highest TP and the lowest FN. Although TCNet achieved a slightly higher mIoU (0.8536), HPA-UNet-LSNet exhibited more balanced performance in terms of false positives and false negatives. The size-stratified results further revealed that the model’s improvement was most evident for small crowns. In the small-target group, Precision, Recall, F1-score, and mIoU increased from 0.6282 ± 0.0240, 0.8772 ± 0.0231, 0.7318 ± 0.0179, and 0.6498 ± 0.0045 for U-Net to 0.6662 ± 0.0043, 0.8877 ± 0.0201, 0.7611 ± 0.0102, and 0.6929 ± 0.0089 for HPA-UNet-LSNet, demonstrating enhanced robustness for sparse and weak-boundary crown targets.
3. **The proposed model offers a practical RGB-based solution for fine-scale crown monitoring in arid regions.** Visual comparisons and Grad-CAM results indicated that HPA-UNet-LSNet generally produced more concentrated responses over crown regions and relatively reduced activation in irrelevant background areas, which aligns with the quantitative evaluation. Additionally, the model maintained a relatively compact structure, with 24.66 M parameters and a model size of 94.09 MB, remaining less complex than several comparison models while achieving competitive accuracy. These results suggest that HPA-UNet-LSNet strikes a useful balance between segmentation performance and model efficiency, making it a practical technical approach for UAV-based monitoring of desert shrub vegetation.

## Supporting information

S1 Appendix Supplementary materials. (PDF)
