# Supplementary material for: HPA-UNet-LSNet: An LSNet-based U-Net with hybrid pooling attention for accurate segmentation of Haloxylon ammodendron crowns from UAV RGB imagery
Source: PLoS One. 2026 Jun 12;21(6):e0350455. doi: 10.1371/journal.pone.0350455 (PMC13262870; doi:10.1371/journal.pone.0350455)
Supplement: S1 Appendix — (PDF) [file pone.0350455.s001.pdf]

S1 Appendix. Supplementary materials

This appendix provides supplementary materials for the manuscript entitled *HPA-UNet-LSNet: An LSNet-Based U-Net with Hybrid Pooling Attention for Accurate Segmentation of Haloxylon ammodendron Crowns from UAV RGB Imagery.* It includes the shared training hyperparameter settings used for all compared models and the per-image Wilcoxon signed-rank test results.

Training Hyperparameter Settings

The training hyperparameter settings used in this study are summarized in Table 1. Unless otherwise stated, the same hyperparameter configuration was applied to all compared models, including U-Net, Res-U-Net, DeepLabv3+, SegFormer, TCNet, LS-U-Net, and HPA-UNet-LSNet. Specifically, all models were trained using the same batch size, optimizer, learning-rate setting and schedule, loss function, and number of training epochs. No model-specific hyperparameter exceptions were used. For each ablation setting, three random seeds (11, 12, and 13) were adopted, and the reported quantitative results represent the averages over the three runs.

Table 1. Shared training hyperparameter settings used for all compared models.

Category	Setting
Input patch size	640×640
Training output classes	1 foreground class (*Haloxylon ammodendron* crown)
Evaluation classes	2 classes (crown and background)
Random seeds	11, 12, 13
Training epochs	300
Training strategy	Freeze–unfreeze training
Freeze stage	Epochs 0–50
Unfreeze stage	Epochs 51–300
Batch size	4
Optimizer	AdamW
Initial learning rate	1×10^{-4}
Minimum learning rate	1×10^{-6}
Learning-rate schedule	Cosine annealing
Loss function	Dice loss
Instance IoU threshold	0.5

Note: The hyperparameter settings listed in this table were applied uniformly to all compared models, including U-Net, Res-U-Net, DeepLabv3+, SegFormer, TCNet, LS-U-Net, and HPA-UNet-LSNet. No model-specific hyperparameter exceptions were used.

During training, each model predicted one foreground class (*Haloxylon ammodendron* crown), whereas during evaluation the segmentation results were assessed as a binary problem with two classes: crown and background.

Statistical Significance Test of Per-Image Results

A paired Wilcoxon signed-rank test was conducted on per-image results from the test set to compare the baseline U-Net and HPA-UNet-LSNet. For this analysis, per-image metrics were computed using the representative checkpoint selected for each model, which was the checkpoint whose validation performance was closest to the mean result across the three runs. This approach ensured that the statistical comparison reflected typical model performance rather than selectively optimal performance.

It is important to note that the IoU values used in this analysis refer to pixel-level IoU computed for each image, rather than the instance-level IoU threshold (0.5) used for crown matching. Therefore, the statistical

test evaluates differences in segmentation quality at the image level. The corresponding results are provided in Table 2.

Table 2. Per-image Wilcoxon signed-rank test results for U-Net and HPA-UNet-LSNet.

Metric	U-Net	HPA-UNet-LSNet	Wilcoxon statistic	p-value
Pixel-level IoU	0.8227 ± 0.0728	0.8455 ± 0.0684	3827.5	< 0.001
Precision	0.8407 ± 0.1465	0.9179 ± 0.1107	1160.5	< 0.001
Recall	0.9201 ± 0.0994	0.9402 ± 0.0833	788.5	< 0.001
F1-score	0.8732 ± 0.1152	0.9259 ± 0.0883	1393.0	< 0.001

Note: The values for U-Net and HPA-UNet-LSNet are reported as the mean ± standard deviation of per-image results on the test set. IoU refers to the pixel-level Intersection over Union computed for each image. These per-image metrics were obtained from the representative checkpoint selected for each model, rather than from an average over multiple runs.
